# Personalized Pathogenicity Assessment of *RPE65* Gene Mutations Using Patient-Specific hiPSC-Derived Retinal Pigment Epithelium Model

**DOI:** 10.3390/ijms27135643

**Published:** 2026-06-23

**Authors:** Ke Ye, Suai Zhang, Ping Xu, Xiaojing Song, Yuan Wang, Xiufeng Zhong

**Affiliations:** State Key Laboratory of Ophthalmology, Zhongshan Ophthalmic Center, Sun Yat-sen University, Guangdong Provincial Key Laboratory of Ophthalmology and Visual Science, Guangzhou 510060, China; boji_0122@hotmail.com (K.Y.); zhangsuai@gzzoc.com (S.Z.); xuping7@mail2.sysu.edu.cn (P.X.); songxiaojing@gzzoc.com (X.S.); wangy2583@mail2.sysu.edu.cn (Y.W.)

**Keywords:** Leber’s congenital amaurosis, *RPE65*, retinal pigment epithelium, stem cells, vitamin A cycle, gene therapy

## Abstract

*RPE65*, an isomerohydrolase expressed in retinal pigment epithelium (RPE), is critical for the visual cycle. More than 115 missense variants of the *RPE65* gene have been associated with Leber’s congenital amaurosis (LCA), a severe childhood retinal dystrophy. Due to high genetic heterogeneity, the variant-specific pathogenic mechanisms remain largely uncharacterized. In this study we focus on an LCA patient carrying compound heterozygous *RPE65* variants (c.200T > G, c.430T > C), aiming to dissect the mechanistic/functional basis of mutated protein-driven retinal degeneration and evaluate gene therapy-mediated restoration using patient-specific hiPSCs-RPE (iRPE). Transient overexpression of wild-type/mutant *RPE65* in HEK293T cells showed both variants markedly destabilize the *RPE65* protein through the autophagosome–lysosome degradation pathway and its isomerohydrolase activity required for the retinoid visual cycle. We further established a patient-specific iRPE platform suitable for enzymatic activity analysis. Characterization of patient-specific iRPE cells revealed those compound heterozygous variants did not compromise iRPE morphology, most gene expression, or core canonical physiological features of iRPE. However, they significantly downregulate endogenous RPE65 protein abundance and dampen enzymatic function. Subsequently, we delivered *RPE65* via adeno-associated viral (AAV) vectors driven by either the ubiquitous CMV promoter or RPE-specific *VMD2* promoter into patient iRPE to validate therapeutic potency, and verified that exogenous *RPE65* supplementation effectively restores deficient isomerohydrolase activity in this disease model. Collectively, this work elucidates the variant-specific pathogenesis of *RPE65*-associated LCA and preliminarily assesses the efficacy of gene augmentation, providing preclinical experimental evidence to support the referral of this patient for clinical *RPE65* gene replacement therapy.

## 1. Introduction

Leber congenital amaurosis (LCA) is the earliest and most severe inherited retinal disorder in children [1]. Patients experience a loss of photoreceptor cells within the retina, leading to congenital blindness by one year after birth [2]. Given the limited regenerative potential of mature retinal cells, available therapeutic interventions remain scarce. LCA is predominantly inherited in an autosomal recessive pattern, with a large panel of disease-causing gene variants (RetNet: https://retnet.org, accessed from 2022 to 2025). Of these disease-associated genes, the *RPE65* gene (retinal pigment epithelium-specific 65) is one of the important genes linked to LCA pathogenesis [3].

*RPE65*, an isomerase expressed in retinal pigment epithelial (RPE) cells, is indispensable for the vitamin A cycle (visual cycle). It catalyzes the conversion of all-trans retinyl ester (at-RE) into 11-cis retinol (11-cis ROL) [4]. 11-cis ROL is subsequently converted into 11-cis retinaldehyde (11-cis RAL). This metabolite travels to photoreceptors and conjugates with opsin to enable phototransduction from light stimuli into electrical signals. At-RE is a subsequent product of all-trans retinaldehyde (at-RAL) produced in photoreceptor cells upon receiving light signals. After releasing at-RAL, photoreceptor proteins lose their ability to convert light signals and must recombine with 11-cis RAL to regain physiological activity [5]. Therefore, pathogenic variants in *RPE65* leading to LCA may involve multiple pathological mechanisms: compromised enzymatic activity, cytotoxicity from the accumulation of at-RE and at-RAL in the visual cycle and impaired degradation of misfolded mutant RPE65 protein. These abnormalities drive the progressive degeneration of RPE and photoreceptor cells and ultimately lead to irreversible blindness [6]. To date, more than 162 disease-associated variants have been identified in the *RPE65* gene, with divergent pathogenic potency and molecular mechanisms dictated by variant location and type. While genetically engineered animal models serve as indispensable research tools for the study of *RPE65*-related LCA, such models frequently fail to fully replicate human disease phenotypes [7]. Therefore, developing patient-specific human-induced pluripotent stem cell (hiPSCs) models customized to individual *RPE65* variants represents a powerful platform to dissect underlying disease mechanisms [8].

hiPSC-derived RPE (iRPE) recapitulates the naïve RPE characteristics and has been widely adopted both as a cell resource for regenerative medicine and an experimental platform for preclinical gene therapy research [9,10]. For *RPE65*-related LCA, preclinical studies using animal models have accelerated the translation of adeno-associated virus (AAV)-mediated gene augmentation therapy into clinical trials [11,12], culminating in the FDA approval of the first gene augmentation therapy drug [13,14,15,16]. However, a subset of treated patients fails to attain satisfactory vision improvement with this high-cost intervention [17]. Because therapeutic outcomes observed in animal models often cannot be reliably recapitulated in human subjects [18], personalized in vitro models offer great potential for predicting therapeutic efficacy prior to proceeding with a gene therapy surgical procedure—specifically in confirming functional recovery and optimizing effective doses. Moreover, few studies have systematically quantified changes in RPE65 isomerohydrolase activity following gene supplementation in either patient-derived cells or animal tissues, even though AAV delivery is presumed to directly rescue enzymatic function [18,19]. Therefore, establishing a robust quantitative assay for measuring isomerase activity using patient-specific models in vitro will help to formulate more rational strategies and accelerate translational drug development. Nevertheless, the application of patient-specific iRPE to the study of isomerase activity remains limited.

During clinical screening, we have enrolled a patient with LCA harboring compound heterozygous *RPE65* variants c.200T > G (L67R) and c.430T > C (Y144H), from whom patient-specific hiPSCs were successfully generated via somatic cell reprogramming [8]. To dissect the pathogenicity of these two variants, we combined transient transfection in HEK293T cells and patient-specific iRPE models to characterize how these substitutions alter RPE65 protein stability and enzymatic activity, which may be the underlying mechanisms contributing to disease onset. Moreover, we compared the in vitro therapeutic performance of AAV-based gene augmentation controlled by distinct promoters by quantifying restored RPE65 protein abundance and isomerohydrolase activity in patient-specific iRPE. Notably, this study is the first to quantitatively assess visual cycle-related enzymatic function of endogenous RPE65 using patient-derived tissue, which not only clarifies variant-specific pathogenic cascades for *RPE65*-associated LCA but also provides a valuable platform for preclinical evaluation of AAV gene replacement across inherited retinal disorders.

## 2. Results

### 2.1. Instability of the RPE65 Protein Bearing c.200T > G and c.430T > C Pathogenic Variants

**Figure 1:**
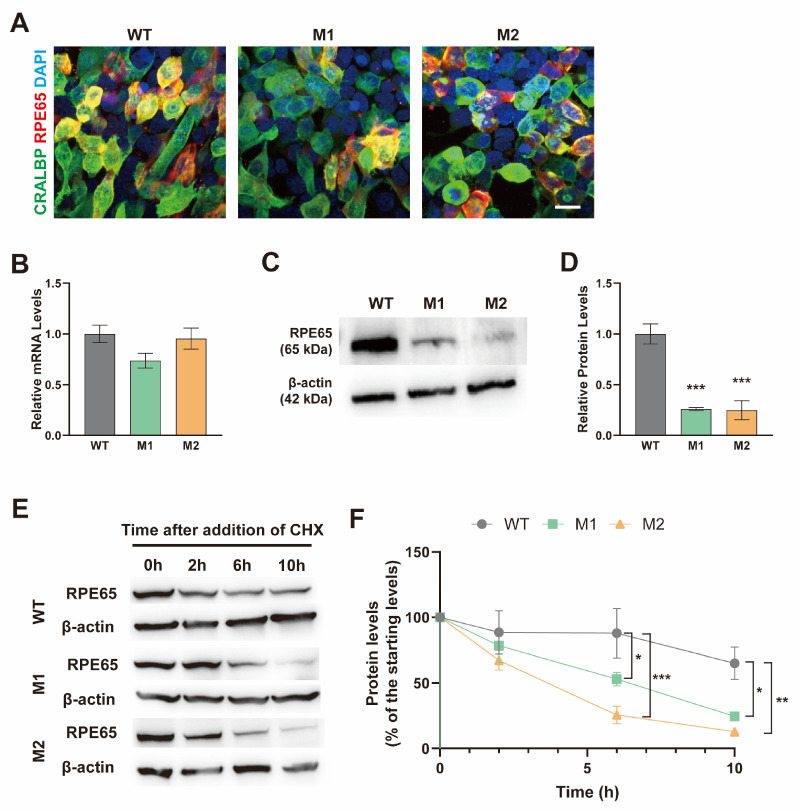
The c.200T > G and c.430T > C variants induce the destabilization of RPE65 protein. (**A**) Representative immunofluorescence images for RPE65 and CRALBP in HEK293T cells transfected with plasmids construct encoding WT or mutant *RPE65*. Scale bar = 20 μm. (**B**) Quantification of RNA level of *RPE65* in HEK293T cells transfected with WT or mutant *RPE65*. Data represent mean ± SEM. (**C**) Western blot analysis of RPE65 protein in HEK293T cells transfected with WT or mutant *RPE65*. (**D**) Quantification of protein level of RPE65 in HEK293T cells transfected with WT or mutant *RPE65*. Data represent mean ± SEM. *** *p* < 0.001 vs. WT. (**E**) Western blot analysis of WT and mutant RPE65 protein in HEK293T cells after treatment of CHX at distinct time. (**F**) Quantification of protein level of WT and mutant RPE65 in HEK293T cells following CHX treatment at distinct time. * *p* < 0.05, ** *p* < 0.01 and *** *p* < 0.001 vs. WT.

An in vitro visual cycle system was built in HEK293T cells via co-transfection with pcDNA3.1-*RPE65*-*CRALBP* and pcDNA3.1-*LRAT* plasmids. To dissect the pathogenic effects of the c.200T > G (M1) and c.430T > C (M2) variants, pcDNA3.1-*RPE65*^WT^, pcDNA3.1-*RPE65*^200T>G^ and pcDNA3.1-*RPE65*^430T>C^ were designed and individually transfected into HEK293T cells (Figure S1A,B). Prior to transfection with *RPE65*-encoding plasmids, empty vector controls were transfected to verify the absence of endogenous *RPE65* expression in HEK293T cells, consistent with earlier published findings (Figure S2) [20]. Immunofluorescence cytochemistry (IFC) confirmed the expression of CRALBP and RPE65 following plasmids delivery (Figure 1A). Notably, Western blot (WB) quantification demonstrated that both M1 and M2 variants markedly lowered RPE65 protein abundance without altering *RPE65* mRNA transcription (Figure 1B–D). We hypothesized that compromised protein stability was altered. To validate this hypothesis, a cycloheximide (CHX) chase assay was performed to quantify the half-life of the mutant protein. Cells transfected with three types of plasmids were treated with cycloheximide (CHX) in 10 µM and cell lysates were collected at 0, 2, 6 and 10 h post-treatment for immunoblotting of RPE65. As a result, compared with the WT RPE65 protein (half-life > 10 h, consistent with previous report [21]), M1 or M2 substitution significantly accelerated the degradation of the RPE65 protein, shortening protein half-lives to below 7 h (M1) and 5 h (M2), respectively (Figure 1E,F). Collectively, these data indicate that the two single-nucleotide substitutions destabilize the mutant RPE65 protein.

### 2.2. Autophagosome–Lysosome-Dependent Degradation of RPE65 Carrying c.200T > G and c.430T > C Variants

**Figure 2:**
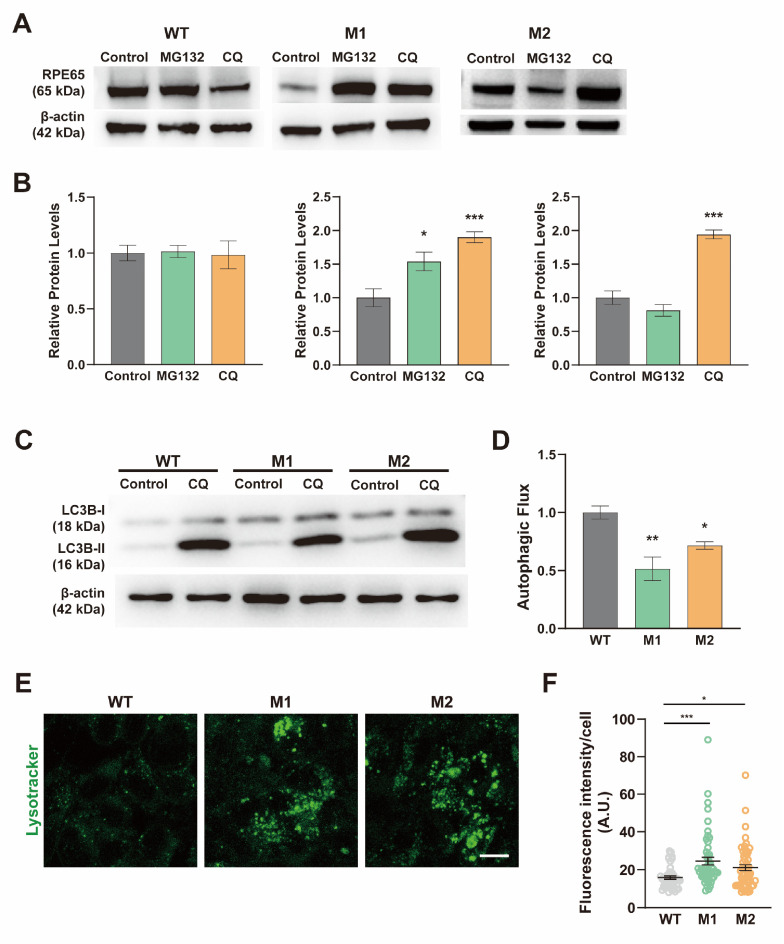
The destabilization of mutant RPE65 is mediated by the autophagosome–lysosomal degradation pathway. (**A**) Western blot analysis for RPE65 in WT and mutant *RPE65* in HEK293T cells following treatment with MG132 or CQ. (**B**) Quantification of protein level of WT and mutant RPE65 in HEK293T cells following treatment with MG132 or CQ. * *p* < 0.05, *** *p* < 0.001 vs. WT. (**C**) Western blot analysis for LC3B in CQ-treated HEK293T cells expressing WT or mutant *RPE65*. (**D**) Quantification of autophagic flux in HEK293T cells expressing WT or mutant *RPE65*. * *p* < 0.05, ** *p* < 0.01 vs. WT. (**E**) Representative images after Lysotracker staining for lysosomes in HEK293T cells expressing WT or mutant *RPE65*. Scale bar = 10 μm. (**F**) Quantification of Fluorescence intensity after staining in 47 (WT), 52 (M1) and 56 (M2) cells. Data represent mean ± SEM. * *p* < 0.05, *** *p* < 0.001 vs. WT.

To explore the degradation pathways responsible for the mutant RPE65 protein, transfected cells were treated with chloroquine (CQ, an autophagic inhibitor, 30 µM) or MG132 (a proteasome inhibitor, 10 µM) for 16 h. While MG132 treatment restored the protein level of RPE65^T200G^, CQ treatment profoundly blocked the degradation of the mutant RPE65 protein (Figure 2A,B), which indicated that the autophagosome–lysosomal pathway was mainly involved in the breakdown of these two mutant RPE65 proteins.

We further assessed the autophagic flux by analyzing LC3B accumulation in HEK293T expressing *RPE65*^WT^, *RPE65*^T200G^ or *RPE65*^T430C^. CQ treatment was performed to induce LC3B turnover, which could be used to explore the transit of LC3B though the autophagic pathway [22]. As a result, quantitative comparison of LC3B levels with or without CQ treatment revealed that autophagic flux was significantly suppressed in cells expressing either M1 or M2 variants (Figure 2C,D). In parallel, LysoTracker staining was conducted in HEK293T cells transfected with WT or mutant *RPE65* plasmids following a previously described protocol [23]. Quantitative fluorescence analysis revealed markedly enhanced LysoTracker signal intensity in mutant *RPE65*-expressing cells (Figure 2E,F), suggestive of elevated endosomal and lysosomal content upon mutant protein expression. Taken together, these results demonstrate that the autophagosome–lysosomal pathway is mainly responsible for the degradation of RPE65^T200G^ and RPE65^T430C^ proteins.

### 2.3. Functional Impairment of Isomerohydrolase Activity for RPE65 Carrying c.200T > G and c.430T > C Variants

**Figure 3:**
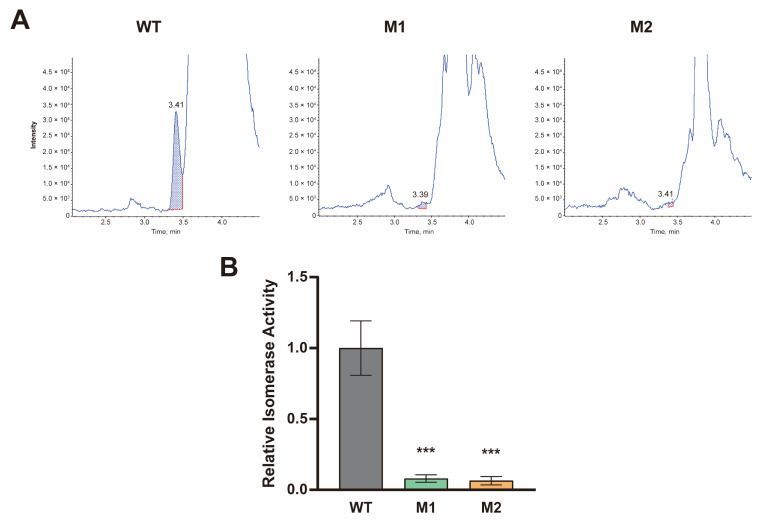
The c.200T > G and c.430T > C variants abolish isomerohydrolase activity in visual cycle. (**A**) Representative LC-MS chromatogram of 11-cis ROL extracted from at-ROL-treated HEK293T cells expressing WT or mutant *RPE65*. (**B**) Quantification of 11-cis ROL extracted from at-ROL-treated HEK293T cells expressing WT or mutant *RPE65*. *** *p* < 0.001 vs. WT.

As an essential isomerohydrolase governing the retinoid visual cycle, RPE65 catalyzes the conversion of all-trans retinyl ester to 11-cis retinol (11-cis ROL). To determine whether disease pathogenesis stems from compromised enzymatic function, we quantified the production of 11-cis ROL after all-trans retinol (at-ROL) treatment in HEK293T expressing *RPE65*^T200G^ or *RPE65*^T430C^ variants using liquid chromatography-mass spectrometry (LC-MS). Prior to sample detection, we established a separation condition to resolve 11-cis ROL from at-ROL using the standard of each compound. Under optimized conditions, 11-cis ROL exhibited a retention time between 3.4 and 3.6 min, whereas at-ROL was after 3.9 min (Figure S3). Then, cell lysates of HEK293T expressing WT or mutant *RPE65* after at-ROL treatment for 16 h were collected to compare the enzymatic activity. LC-MS results revealed greater yield of 11-cis ROL in WT *RPE65*-expressing cells (Figure 3A,B), demonstrating that both substitutions abolish endogenous isomerohydrolase activity, most likely as a downstream consequence of accelerated mutant protein degradation.

### 2.4. Generation and Functional Characterization of Polarized Patient-Specific iRPE Cells Harboring Compound Heterozygous c.200T > G and c.430T > C Variants Using 2D Differentiation Protocol

To further dissect the pathological mechanisms linking these compound heterozygous variants to vision impairment in a patient with LCA, we generated patient-specific iRPE cells to compare the difference of an iRPE control with WT *RPE65*. Meanwhile, a *RPE65* knockout hiPSC line was established from BC1 hiPSCs using CRISPR/Cas9 gene-editing technology to serve as a positive control (Figure S4).

**Figure 4:**
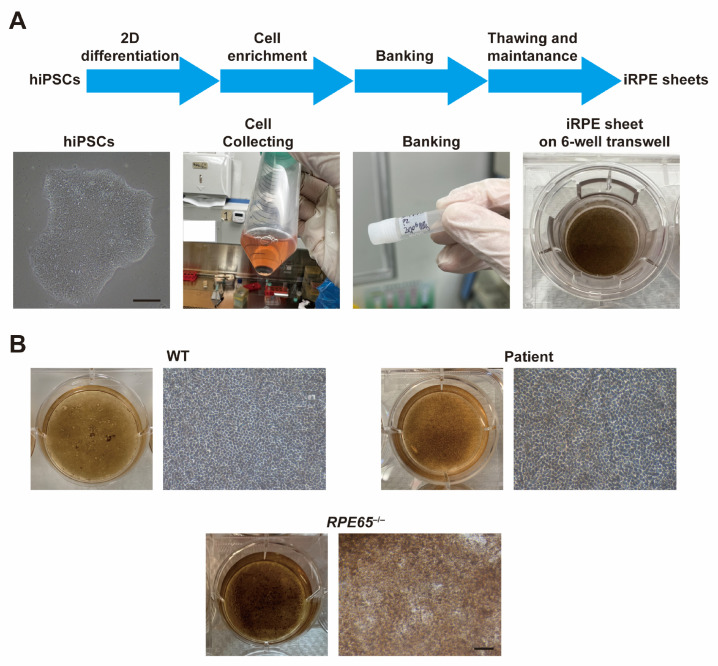
A patient-specific iRPE model suitable for LC-MS analysis is established. (**A**) Schematic illustration of the strategy for LC-MS analysis using iRPE cells. Scale bar = 100 μm. (**B**) Macroscopic and microscopic images of WT, patient-specific and *RPE65*^−/−^ iRPE cells post differentiation. Scale bar = 50 μm.

**Figure 5:**
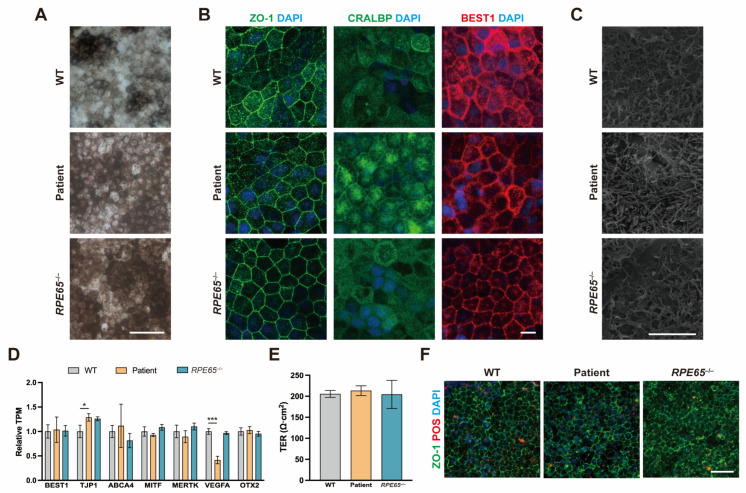
Large scale of polarized iRPE cells from WT, patient-specific (compound heterozygous c.200T > G and c.430T > C variants in *RPE65*) and *RPE65*^−/−^ hiPSCs are generated and phenotypic-characterized. (**A**) Representative microscopic images of WT, patient-specific and *RPE65*^−/−^ iRPE cells on Transwell inserts. Scale bar = 50 μm. (**B**) Representative immunofluorescence images for ZO-1, CRALBP and *BEST1* in WT, patient-specific and *RPE65*^−/−^ iRPE cells. Scale bar = 10 μm. (**C**) Representative SEM images of WT, patient-specific and *RPE65*^−/−^ iRPE cells. Scale bar = 5 μm. (**D**) Relative TPM of RPE-relative genes among WT, patient-specific and *RPE65*^−/−^ iRPE cells. * *p* < 0.05, *** *p* < 0.001 vs. WT. (**E**) TER values of WT, patient-specific and *RPE65*^−/−^ iRPE cells. (**F**) Representative immunofluorescence images for ZO-1 in WT, patient-specific and *RPE65*^−/−^ iRPE cells following exposure of CM-Dil labeled photoreceptor outer segments (POS). Scale bar = 50 μm.

According to previous reports, large batches of polarized iRPE cells are required for an in vitro retinol-processing experiment [24]. Accordingly, three types of hiPSCs were differentiated into iRPE cells by 2D differentiation protocol and further cultured on 6-well Transwell inserts (Figure 4A) [25]. After 35 days of differentiation, all three hiPSCs were induced into RPE cells in high efficiency by quantification of the pigmented area in the culture plate (Figure 4B and Figure S5). Following 8 weeks of culture on the Transwell insert, three types of iRPE cells were developed into mature RPE monolayers, characterized by canonical polygonal pigmented morphology (Figure 5A) and the expression of signature RPE markers including ZO-1, BEST1 and CRALBP (Figure 5B). Scanning electron microscopy (SEM) observation showed normal apical processes in three types of RPE monolayers (Figure 5C).

Transcriptomic profiling using RNA-seq was performed to quantify the transcriptional divergence among the three groups of iRPE cells. The correlation analysis revealed that the expression pattern of iRPE cells did not significantly change after knockout of *RPE65* (Figure S6A), with only a limited subset of differentially expressed genes (DEGs) detected between WT and *RPE65*^−/−^ iRPE cells (Figure S6B). These results suggest that complete ablation of *RPE65* barely interferes with RPE differentiation and maturation. In patient-specific iRPE cells, modest variations in the expression of several RPE markers were observed, presumably driven by the inherent donor genetic background (Figure 5D). Nevertheless, all three cell groups displayed comparable transepithelial resistance (TER) and phagocytosis capacity (Figure 5E,F). In conclusion, we have successfully established a patient-specific iRPE platform to generate *RPE65*-deficient iRPE, which enables subsequent mechanistic and enzymatic activity investigation of LCA pathogenesis.

### 2.5. Enhanced Degradation of Mutant RPE65 in Patient-Specific iRPE Cells

**Figure 6:**
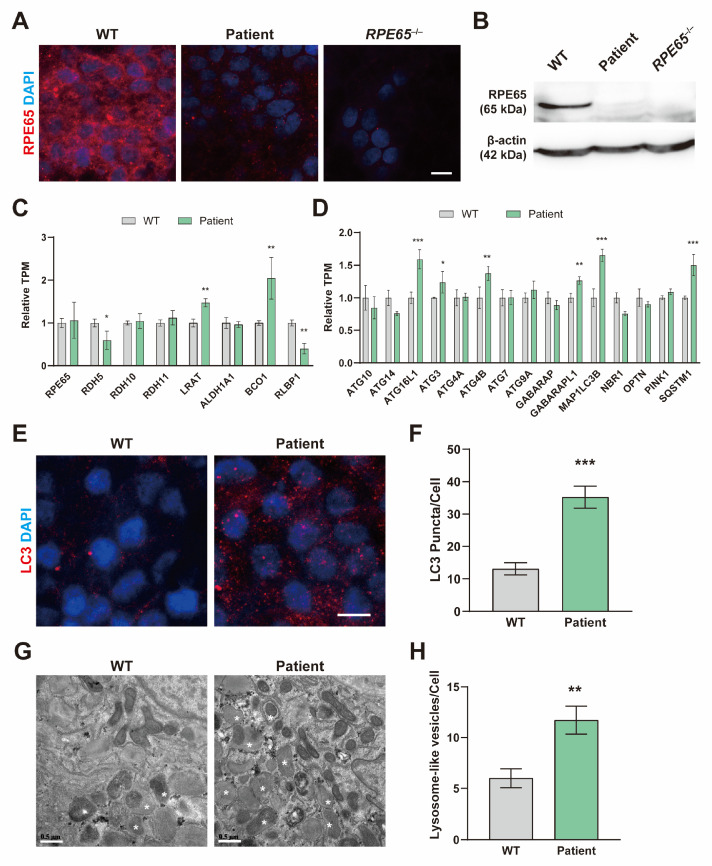
The compound heterozygous c.200T > G and c.430T > C variants in *RPE65* trigger enhanced protein degradation of RPE65 in patient-specific iRPE cells. (**A**) Representative immunofluorescence images for RPE65 in WT, patient-specific and *RPE65*^−/−^ iRPE cells. Scale bar = 10 μm. (**B**) Western blot analysis of RPE65 in WT, patient-specific and *RPE65*^−/−^ iRPE cells. (**C**) Relative TPM of visual cycle-relative genes in WT and patient-specific iRPE cells. * *p* < 0.05, ** *p* < 0.01 vs. WT. (**D**) Relative TPM of autophagosome–lysosomal pathway-relative genes in WT and patient-specific iRPE cells. * *p* < 0.05, ** *p* < 0.01 and *** *p* < 0.001 vs. WT. (**E**) Representative immunofluorescence images for LC3B in WT and patient-specific iRPE cells. Scale bar = 10 μm. (**F**) Quantification of LC3 puncta per cells in WT and patient-specific iRPE cells. *** *p* < 0.001 vs. WT. (**G**) Representative SEM images of WT and patient-specific iRPE cells. Asterisks indicate the lysosome-like vesicles. Scale bar = 0.5 μm. (**H**) Quantification of lysosome-like vesicles per cells in WT and patient-specific iRPE cells. ** *p* < 0.01 vs. WT.

In the former part, we identified the compromised protein stability of RPE65 carrying c.200T > G or c.430T > C variants. Next, we sought to clarify whether these compound heterozygote *RPE65* substitutions trigger accelerated protein turnover in patient-derived iRPE cells. Both IFC and WB analysis for RPE65 demonstrated markedly reduced RPE65 protein abundance in both patient-specific iRPE and *RPE65*^−/−^ iRPE relative to WT controls (Figure 6A,B). Given the comparable transcriptomic level of *RPE65* between patient-specific iRPE cells and WT iRPE cells (Figure 6C), the lower protein level of RPE65 most likely arises from the destabilization of the mutant protein.

Our earlier analysis validated the lysosome–autophagy pathway associated with degradation of the RPE65 protein with c.200T > G or c.430T > C mutations (Figure 2). Consistently, RNA-seq analysis of patient-specific iRPE cells also confirmed the different expression pattern of autophagy pathway-related genes, including the elevated mRNA levels of *MAP1LC3B* (encoding LC3B) and *SQSTM1* (encoding p62) (Figure 6D). IFC and WB analysis of LC3B showed comparable total LC3 abundance but accumulation of LC3 puncta in patient-specific iRPE cells (Figure 6E,F and Figure S7), indicative of activated autophagosomal turnover targeting mutant RPE65 [26]. Furthermore, more lysosome-like vesicles were observed in patient-specific iRPE cells by transmission electron microscopy (TEM) analysis (Figure 6G,H). Therefore, these results suggest that the lower level of RPE65 in patient-specific iRPE cells is associated with the lysosome–autophagy-related protein degradation.

### 2.6. Functional Restoration of Patient-Specific iRPE Cells in Isomerase Activity with AAV-Mediated Gene Augmentation Therapy

**Figure 7:**
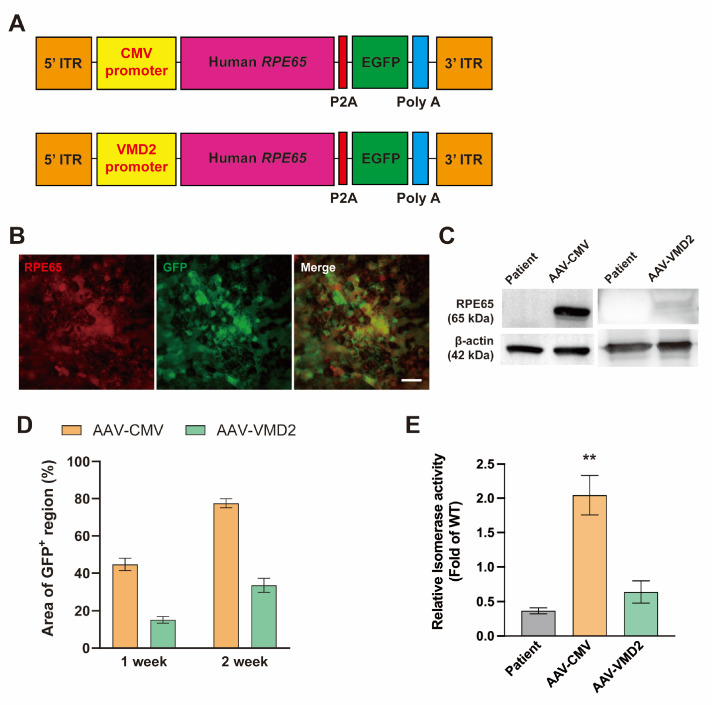
AAV-mediated gene supplementation restores the protein expression and enzymatic function in patient-specific iRPE cells. (**A**) Schematic diagram of two AAV vectors carrying full-length human *RPE65* expression cassette by either CMV or *VMD2* promoters. (**B**) Representative immunofluorescence images for *RPE65* in patient-specific iRPE cells after transduction of AAV-*VMD2* vector (red, RPE65; green, GFP). Scale bar = 20 μm. (**C**) Western blot analysis of RPE65 in patient-specific iRPE cells after transduction of AAV-*VMD2* or AAV-CMV vectors. (**D**) Quantification of GFP-positive area in patient-specific iRPE cells at 1 and 2 weeks post AAV transduction. (**E**) Quantification of 11-cis ROL extracted from patient-specific iRPE cells transduced with AAV-*VMD2* or AAV-CMV vectors after at-ROL treatment. ** *p* < 0.01 vs. patient.

Subretinal injection of AAV carrying a normal copy of the human *RPE65* cDNA to RPE cells has been demonstrated to restore visual function in both preclinical animal models and human patients with retinal degeneration. AAV-mediated *RPE65* expression is commonly driven by either the CMV ubiquitous promoter or the RPE-specific *RPE65* promoter [14]. Here, we evaluated the therapeutic efficiency of AAV2.7m8 expressing *RPE65* cDNA under the control of either the CMV or *VMD2* promoter. The *VMD2* gene encodes BEST1, another RPE-specific transmembrane protein that is localized at the basolateral plasma membrane. The *VMD2* promoter confers superior RPE specificity and transcriptional efficiency compared with the *RPE65* promoter [27]. We constructed two vectors containing the full length of the human *RPE65* expression cassette along with the enhanced green fluorescence protein (eGFP) driven by distinct promoters (Figure 7A).

After six weeks of culture, patient-specific iRPE cells were transduced with AAV vectors and analyzed after 2 weeks. To determine the suitable viral dosage, iRPE cells were infected with the AAV-*VMD2*-*RPE65* vector (AAV-*VMD2*) with a different multiplicity of infection (MOI), including 0, 1 × 10^4^, 3 × 10^4^ and 1 × 10^5^ viral genome copies per cell (GC/cell). Two weeks after transduction, protein expression of RPE65 was up-regulated at MOI of 3 × 10^4^ and 1 × 10^5^ GC/cell (Figure S8A) without obvious cell apoptosis (Figure S8B). Both GFP and RPE65-positive signals were identified in transduced patient-specific iRPE cells (Figure 7B). Accordingly, the moderate and safe dosage of 3 × 10^4^ GC/cell was selected for subsequent function validation.

Furthermore, we compared the rescue efficiency between the AAV-CMV-*RPE65* vector (AAV-CMV) and AAV-*VMD2*. As a result, iRPE cells with AAV-CMV transduction showed higher RPE65 protein level than cells with AAV-*VMD2* transduction (Figure 7C). Live-cell fluorescence imaging further confirmed a larger GFP-positive area in iRPE cells with AAV-CMV transduction (Figure 7D and Figure S8C). Functional assessment of retinoid isomerohydrolase activity revealed only a slight restoration of enzymatic activity in iRPE cells with AAV-*VMD2* transduction, while AAV-CMV treatment achieved a better function rescue (Figure 7E and Figure S9). Collectively, these findings demonstrate that the patient-specific iRPE platform serves as a robust and reproducible in vitro system for preclinical evaluation of AAV-based gene therapy efficacy for inherited retinal diseases.

## 3. Discussion

In this study, we investigated the pathogenic mechanisms underlying *RPE65* c.200T > G and c.430T > C variants. Transient overexpression of mutant *RPE65* in HEK293T showed that these substitutions destabilize the RPE65 protein, thereby impairing retinoid visual cycle function. This turnover is predominantly driven by the activated autophagy/lysosomal-related pathways. To further characterize the pathogenic phenotypes of compound heterozygous *RPE65* mutations, we applied a 2D differentiation protocol to generate large-scale, polarized iRPE monolayers on 6-well Transwell inserts, which were sufficiently robust for subsequent LC-MS metabolic analysis. Consistent with the overexpression cell model, patient-specific iRPE cells exhibited key disease phenotypes, including reduced RPE65 protein stability and compromised isomerohydrolase activity. Notably, AAV-mediated gene augmentation therapy restored RPE65 protein abundance and rescued enzymatic activity in patient-specific iRPE cells. The patient-specific iRPE platform not only enables in-depth mechanistic exploration of inherited retinal disease pathogenesis but also provides a reliable and reproducible in vitro system for preclinical evaluation of therapeutic gene-editing strategies.

The minimal visual cycle using HEK293T with transient expression of *RPE65* represents a widely adopted tool for evaluating the pathogenicity of diverse *RPE65* point mutations and identifying variants that cause severe impairment of isomerohydrolase activity [4,20,21,28,29]. Here, we comprehensively evaluated the protein expression level and enzymatic activity of RPE65 carrying c.200T > G and c.430T > C variants, which have not been functionally characterized to date. The c.200T > G missense variant has been validated as a pathogenic mutation in previous studies [30], while the c.430T > C variant was first reported in 2013 [31]. Nevertheless, the mechanism by which these two mutations induce vision loss remains poorly understood. This research reveals that both variants trigger excessive degradation of the mutant RPE65 protein through the lysosome-dependent autophagic pathway. The loss of the RPE65 protein further abolished the isomerohydrolase function, which is a critical pathological driver of LCA pathogenesis. A recent study has developed a high-throughput assay based on a similar model to assess the pathogenicity of *RPE65* variants of unknown significance (VUS) but it lacks the exploration of functional activity [32]. Our research also provides a stable and reproducible approach to measure isomerohydrolase activity, which can be broadly applied for the pathogenicity analysis of uncharacterized *RPE65* VUS and the preclinical evaluation of therapeutic efficacy following AAV-based gene therapy.

A major limitation of conventional in vitro visual cycle models is the difficulties in recapitulating complex heterozygous *RPE65* variants with multiple mutation sites. Although patient-specific iRPE models can potentially overcome it, several technical challenges remain to be resolved: (1) the need for a large yield of iRPE cells to quantify the amount of retinol by LC-MS analyses, which requires a highly efficient differentiation and expansion protocol [24,33,34]; (2) sufficient iRPE cells should be cultivated under polarized conditions to sustain physiological functionality [35]. To overcome these limitations, we optimize a 2D differentiation protocol based on small-molecules treatment. This protocol enables the generation of approximately 1 × 10^7^ iRPE cells from a single well of a 6-well plate in one batch (WT: (1.27 ± 0.03) × 10^7^ cells; Patient: (1.16 ± 0.08) × 10^7^ cells; *RPE65*^−/−^: (1.43 ± 0.18) × 10^7^ cells for three batch). Furthermore, iRPE cells were further cultured in a 6-well Transwell insert, a well-established method to generate polarized iRPE cells [36]. All iRPE cells exhibited typical features of the RPE monolayer, including the expression of mature RPE markers and physiological functions. Importantly, this standardized culture pipeline is compatible with visual cycle activity evaluation, providing a robust and practical platform for retinal disease modeling, preclinical drug assessment, and therapeutic strategy validation for inherited retinal disorders.

Comparisons among WT, *RPE65*^−/−^ and patient-specific iRPE cells revealed that the reduced/absent RPE65 protein level does not impair RPE development or most cellular functions independent of the visual cycle. Consistent with our finding, animal models have demonstrated that genetic ablation of *RPE65* does not disrupt RPE development in dogs and mice but leads to the accumulation of lipid-like inclusions after birth [7,37,38,39]. In line with these observations, our previous work using human retinal organoids also confirms that this compound heterozygous *RPE65* mutation does not impact the development of the neural retinal [8]. Collectively, these findings indicate that *RPE65* mutation-driven retinal degeneration is primarily attributed to the loss of enzymatic activity in the visual cycle. To validate this hypothesis, LC-MS was used to assess the conversion enzyme activity in patient-specific iRPE cells. Although previous studies have validated the capability of hiPSCs-derived iRPE cells to execute visual cycle functions in vitro [19], this study represents the first analysis to characterize visual cycle dysfunction in patient-specific iRPE cells from LCA patients.

Since AAV2-7m8 has been found to have a superior transduction efficiency in hiPSC-derived retinal organoids (ROs) and RPE [40], we utilized this serotype to compare the functional restoration efficacy of AAV vectors driven by a ubiquitous promoter and an RPE-specific promoter. Most previous studies have focus on the therapeutic performance of AAV with a CMV or CAG promoter [41,42,43], while a head-to-head comparison between ubiquitous and RPE-restricted promoters remains insufficiently explored. Although the *RPE65* promoter has been applied in clinical trial [14], we select the *VMD2* promoter because of *VMD2* promoter’s superior regulatory efficiency in animal models [27]. Our functional validation demonstrated that both AAV-CMV and AAV-*VMD2* successfully transduced patient-specific iRPE cells, whereas AAV-CMV yielded significantly better functional rescue than AAV-*VMD2*. One potential reason is that the CMV promoter shows the superiority in driving transgene expression [44,45], because this ubiquitous promoter originates from its viral-derived dense enhancer array packed with binding motifs for ubiquitously expressed transcription factors [46,47]. In addition, only a truncated human *VMD2* promoter (−585/+38 bp) was used due to the packaging capacity constraint of rAAV [48]. Furthermore, the activity of the *VMD2* promoter relies on endogenous *BEST1* expression. According to previous studies, stable and uniform *BEST1* expression is only achieved after long-term culture of iRPE cells for up to 12 months [49]. The relatively weak transcriptional activity of the *VMD2* promoter and heterogeneous *BEST1* expression in iRPE cells collectively resulted in only marginal restoration of isomerohydrolase activity in AAV-*VMD2*-transduced patient iRPE cells. Notably, AAV-CMV-treated patient-specific iRPE even exhibited higher enzymatic activity than the WT iRPE control. This phenomenon may be attributed to the abundant at-ROL substrate supplied in the system and supraphysiological *RPE65* expression driven by AAV-CMV, consistent with the substrate- and protein-level-dependent characteristics of RPE65 activity [50,51]. A more precise exploration of the correlation between rescued enzymatic activity and visual function recovery in subsequent studies is critical for defining ideal gene therapy strategies and dosages in clinical practice.

In summary, we established an in vitro visual cycle using HEK293T cells with transient *RPE65* overexpression and verified that the c.200T > G or c.430T > C variants accelerate mutant RPE65 protein degradation predominantly through autophagy/lysosomal-related pathways, thereby abolishing the enzymatic activity essential for the visual cycle. A personalized in vitro model using patient-specific iRPE cells is built to validate that the compound heterozygous *RPE65* c.200T > G and c.430T > C mutations markedly reduce the stability of the RPE65 protein, ultimately disrupting visual cycle function. This process may also be associated with the activation of lysosomal-related pathways. Additionally, we systematically compared the functional rescue efficacy of AAV vectors mediated by the ubiquitous CMV promoter and the RPE-specific *VMD2* promoter in patient-derived iRPE cells. Our findings demonstrate that the patient-specific iRPE model serves as a reliable tool for evaluating the preclinical efficacy of AAV-mediated gene therapy for inherited retinal disorders.

## 4. Materials and Methods

### 4.1. Cell Culture

HEK293T cells were purchased from Cell Bank, Type Culture Collection, Chinese Academy of Sciences (SCSP-5293, Shanghai, China). Cells were cultured in DMEM supplemented with 10% fetal bovine serum (FBS) and 1% penicillin-streptomycin (P/S, Gibco, Grand Island, NY, USA). In some experiments, cells were treated with 10 µM cycloheximide (MCE, Monmouth Junction, NJ, USA), 10 µM MG132 (GLPBIO Technology Inc, Montclair, CA, USA) or 30 µM chloroquine (Targetmol, Boston, MA, USA) for a distinct time.

BC1-hiPSCs were a gift from Professor Linzhao Cheng of University of Science and Technology of China (Hefei, China) [52]. Patient-hiPSCs were generated by reprogramming of urine cells from a patient with compound heterozygous *RPE65* variants [8]. hiPSCs were cultured on 6-well plates coated with Matrigel (Corning, Corning, NY, USA) in a mTeSR1 medium (Stem Cell Technologies, Vancouver, BC, Canada) at 5% CO_2_ in a 37 °C incubator. Medium was refreshed every other day. When cells reached 80% confluency, hiPSCs were dissociated with 0.5 mM EDTA (Invitrogen, Grand Island, NY, USA) for passage.

### 4.2. Generation of RPE65^−/−^ hiPSCs

A total of 2–3 highly specific single-guide RNA (sgRNA) sequences targeting critical coding exons (exon 2 or 3) of the human *RPE65* gene were designed online using CRISPOR and inserted into the pX330-U6-Chimeric-BB-CBh-hSpCas9 plasmid (42,230; Addgene, Watertown, MA, USA). The CRISPR/Cas9-sgRNA plasmids were electroporated into BC1-hiPSCs using the Neon Transfection System (Thermo Fisher Scientific, Waltham, MA, USA). All electroporated cells were seeded onto Matrigel-coated 6-well plates and cultured in mTeSR1 containing 10 μM Blebbistatin (Sigma-Aldrich, St Louis, MO, USA). The medium was replaced with mTeSR1 on the next day, and culture was continued until the clones were nearly fused. Next, the medium of mTeSR1 with 1 µg/mL puromycin (Solarbio, Beijing, China) was used to enrich the transfected cells for 3 days. The surviving hiPSC clones were manually picked and seeded onto Matrigel-coated plates. The *RPE65* genotype of each clone was verified by Sanger sequencing. All primers used are listed in Table S1.

### 4.3. RPE Differentiation

RPE differentiation was performed according to a previous report [25]. Briefly, hiPSCs were treated with accutase for 5 min to dissociate into single cells and plated onto iMatrix 511 (Nippi Inc., Tokyo, Japan)-coated 6-well plates at 3.0 × 10^4^ cells/well. During the first 6 days, cells were treated with 100 nM LDN193189 (Stemgent, Beltsville, MD, USA), 500 nM A-83-01 (Wako, Osaka, Japan), 1 μM IWR-1-endo (Stem Cell Technologies, Vancouver, BC, Canada), and 10 μM Y-27632 (APExBio, Houston, TX, USA) in IMDM (Thermo Fisher Scientific, Waltham, MA, USA)/Ham’s F12 (Sigma-Aldrich, St Louis, MO, USA) (1:1) medium supplemented with 10% KnockOut Serum Replacement (KSR, Thermo Fisher Scientific, Waltham, MA, USA), 0.5 mM Monothioglycerol Solution (Wako, Osaka, Japan), 1% chemically defined lipid concentrate (Gibco, Grand Island, NY, USA), and 2 mM L-glutamine (Gibco, Grand Island, NY, USA). For the next 12 days, cells were cultured in IMDM/F12 containing 3 μM CHIR99021 (Stem Cell Technologies, Vancouver, BC, Canada) and 10 μM Y-27632. From day 18, the medium was changed to DMEM/F12 (Gibco, Grand Island, NY, USA) supplemented with 10% KSR, 1% N2 Supplement (Thermo Fisher Scientific, Waltham, MA, USA), and 2 mM L-glutamine. A 10 mM nicotinamide (Stem Cell Technologies, Vancouver, BC, Canada) was supplemented from day 12 to day 24. Between day 24 and 35, the medium was changed to an RPE maintenance medium including 67% high glucose DMEM (Gibco, Grand Island, NY, USA), 29% Ham’s F12, 2% B27 supplement minus vitamin A (Gibco, Grand Island, NY, USA), 2 mM L-glutamate and 1% P/S.

Cryopreserved iRPE cells were re-cultured on iMatrix 511-coated 6-well plates. When they reached confluence, the passage 3 iRPE cells were dissociated and seeded on Transwell inserts (Corning, Corning, NY, USA) at a density of 1 × 10^5^ cells/cm^2^. The cells were cultured in medium containing 50% DMEM/F12 (Gibco, Grand Island, NY, USA), 35% DMEM basic (Gibco, Grand Island, NY, USA), 10% FBS (Gibco, Grand Island, NY, USA), 2% B27 supplement (Gibco, Grand Island, NY, USA), 1% NEAA (Gibco, Grand Island, NY, USA), 1% GlutaMax (Gibco, Grand Island, NY, USA), 0.1% Taurine (Sigma-Aldrich, St Louis, MO, USA) and 1% P/S. After cells grew into confluence in 10 days, the culture medium was switched into RPE maturation medium consisting of 60% DMEM/F12, 37% DMEM basic, 2% B27 supplement, and 1% P/S. Cells were monitored and photographed with an inverted microscope.

### 4.4. Generation of Expression Vectors and Cell Transfection

WT human *RPE65* and *CRALBP* cDNAs were subcloned into the expression vector pcDNA3.1 to construct a pcDNA3.1-*RPE65* + CRALBP vector. Human *LRAT* cDNA was subcloned into the expression vector pcDNA3.1 to construct a pcDNA3.1-*LRAT* vector. Two site-directed mutations were introduced into the *RPE65* ORF using the QuikChange XL site-directed mutagenesis kit (Stratagene, La Jolla, CA, USA). All constructs and mutants were sequenced using an ABI-3730 DNA sequencer (Applied Biosystems, Foster City, CA, USA) to confirm the orientation and accuracy of the ORFs or the mutants introduced. Plasmids were prepared by using the Magen Maxpure plasmid kits (Magen Biotechnology, Guangzhou, China) according to the manufacturer’s protocols. HEK293T cells were co-transfected with pcDNA3.1-*RPE65*-CRALBP and pcDNA3.1-*LRAT* using PEI MAX transfection reagent (Polysciences, Warrington, PA, USA) following the manufacturer’s instruction.

### 4.5. Construction and Transduction of Recombinant Adeno-Associated Viral (rAAV) Vectors

Commercially available rAAV2.7m8-carrying human *RPE65* cDNA driven by a distinct promoter (AAV-CMV-*RPE65* and AAV-*VMD2*-*RPE65*) were produced by VectorBuilder (Guangzhou, China). Six-week cultured iRPE cells were incubated with the two AAV vectors at various multiplicities of infection (MOI) for 18 h. Following transduction, cells were maintained for an additional two weeks before detection of *RPE65* protein levels and enzymatic activity.

### 4.6. RNA Extraction and Quantitative Real-Time PCR (qPCR)

Total RNA was extracted with Trizol regent (Sigma-Aldrich, St Louis, MO, USA) and qualified on Nanodrop 2000 (Thermo Fisher Scientific, Waltham, MA, USA). Genomic DNA removal and reverse transcription were conducted using the RT reagent kit with a gDNA eraser (Takara, Shiga, Japan). Quantitative PCR was performed using SYBR Green PCR Kit (Qiagen, Germantown, MD, USA) on an ABI StepOne Plus Real-Time PCR System (Thermo Fisher Scientific, Waltham, MA, USA). The thermal cycling protocol was set as follows: pre-denaturation at 95 °C for 3 min, followed by 35 cycles consisting of denaturation at 95 °C for 10 s, annealing at 60 °C for 34 s, and extension at 72 °C for 35 s. PCR product quality was monitored via melting curve analysis. GAPDH was used as the internal control. The 2^−ΔΔCt^ method was used to analyze the data. Triplicates were set for each sample in each experiment. All primers sequences are listed in Table S1.

### 4.7. Western Blot (WB)

Cells were lysed with RIPA cell lysis buffer (Beyotime, Shanghai, China), followed by centrifugation at 13,000× *g* for 30 min at 4 °C. The supernatants were collected and total cellular protein concentrations were measured using a BCA protein assay kit (Beyotime, Shanghai, China). Total protein was denatured by boiling in loading buffer (NCM SDS-PAGE Loading Buffer, 5×, NCMbio, Suzhou, China). Equal amounts of protein were separated by SDS-PAGE [(100 mM Tris-HCl, pH 6.8, 4% (*w*/*v*) SDS, 0.2% (*w*/*v*) bromophenol blue, 20% glycerol and 200 mM DTT (dithiothreitol)]. For Western blot analysis, resolved proteins were transferred on a PVDF membrane (Merck-Millipore, Burlington, MS, USA) which was then blocked with 5% BSA in PBS for 1 h and then incubated with the appropriate primary antibodies (overnight at 4 °C, see Table S2 for antibody details) and subsequently probed with secondary HRP-conjugated antibodies (1 h at room temperature). Protein signal was captured by ChemiDoc MP Imaging Systems (Biorad, Hercules, CA, USA). Densitometric analysis was performed using Image Lab software (Version 6.1, Biorad, Hercules, CA, USA).

### 4.8. Immunohistochemistry (IHC)

Immunostaining was performed as described previously [53,54]. Cells cultured on glass coverslips or Transwell inserts (Corning, Corning, NY, USA) were fixed with 4% paraformaldehyde (PFA) for 15 min at room temperature. After permeabilization with 0.1% Triton X-100 (MP Biomedicals, Irvine, CA, USA) made in 10% normal donkey serum (Solarbio, Beijing, China) for 1 h, samples were incubated with primary antibodies (see Table S2 for antibody details) diluted in 20% ADS overnight at 4 °C. Following 3 PBS washings at room temperature, samples were incubated with appropriate Alexa Fluor-conjugated secondary antibody for 1 h in the dark. Following secondary antibodies were removed and PBS washed, DAPI was used for nuclear counter staining. Images were acquired using either a Zeiss LSM 880 confocal microscope (Zeiss, Jena, Germany) or a ZEISS Axio observer 7 microscope (Zeiss, Jena, Germany).

### 4.9. Phagocytosis

The phagocytosis assay was conducted as previously described [53]. Photoreceptor outer segments (POS) were freshly isolated from porcine eyes in a dark room and labeled with CM-Dil (Invitrogen, Grand Island, NY, USA) following manufacturer instructions. iRPE cells after 8 weeks of culture were incubated with the CM-Dil labeled POS at 37 °C or 4 °C (negative control) for 24 h. Subsequently, all samples were washed with PBS thoroughly, fixed with 4% PFA and stained with DAPI. Images were acquired with a ZEISS Axio observer 7 microscope (Zeiss, Jena, Germany).

### 4.10. Transepithelial Electrical Resistance (TER)

The TER of iRPE monolayers was recorded as previously described [55,56]. Briefly, iRPE cells were cultured in iMatrix 511-coated Transwell inserts for 4 weeks. TER was detected using an EVOM2 Volt–Ohm meter (World Precision Instruments, Sarasota, FL, USA). An empty iMatrix 511-coated Transwell insert containing basic DMEM was used as the blank control. To ensure data reliability, the TER value for each sample was measured five times, and then the average value was calculated. The mean TER values (Ω × cm^2^) were obtained by subtracting the value of the blank control from each measurement.

### 4.11. Isomerohydrolase Activity Assay Using HPLC MS/MS (LC-MS) Analysis

All retinoid-related operations were performed under dim red lighting to avoid photodegradation. For enzymatic activity detection in HEK293T cells, 7 μM of all-trans retinol (atROL) was supplemented to the culture medium at 48 h post-transfection. Cells were harvested at 16 h after substrate addition. For function evaluation in iRPE, 8 weeks of iRPE cells were incubated with medium containing 7 μM all-trans retinol for 48 h. The protein expression and its enzymatic activity were measured by Western blot and in vitro activity assays.

Prior to LC-MS analysis, cell pellets (1 × 10^6^ cells per sample) were resuspended in 200 μL of 0.9% NaCl solution, ultrasonicated for 5 min, and centrifuged at 13,000 rpm for 20 min at 4 °C. The supernatant was added at a dose of 100 μL atROL-D4 as internal standard along with 1 mL of n-hexane, vortexed for 3 min, and centrifuged at 13,000× rpm for 5 min at 4 °C. A total of 0.8 mL of the upper phase was collected and blown dry with nitrogen, followed by 150 μL MeOH/H_2_O (80:20) for injection.

Chromatographic separation of 11-cis-retional (11-cis ROL) and atROL was achieved using a Shimadzu LC-20ACXR (Shimadzu, Kyoto, Japan) equipped with an ACE EXCEL-2 PFP-C18 column (2.1 mm × 100 mm × 2.6 μm, Avantor, Radnor, PA, USA) maintained at 35 °C. Solvent A was composed of 100% H_2_O with 0.1% formic acid, and solvent B was 100% acetonitrile (ACN) with 0.1% formic acid and 2 mM ammonium acetate. The mobile phase system consisted of a 3 min linear gradient from 80 to 95% B, held at 95% B for 0.5 min, then immediately returned to 80% B for 1 min to re-equilibrate. The flow rate was 0.3 mL/min with an injection volume of 2 μL.

A Sciex 5500 triple-quadruple mass spectrometer (Sciex, Framingham, MA, USA) with heated electrospray ionization (ESI) was used for analysis. ESI-MS was operated in positive mode with a voltage of 5500 V, a temperature of 500 °C, curtain gas at 35 psi, GS1 at 50 psi, and GS2 at 55 psi. Optimization of MS/MS parameters for all analyses was performed by selecting precursor ions of [M + H] + for 11-cis ROL and atROL, to obtain the mass transition, was selected as 269 → 93 for 11-cis-retional and atROL 273 → 93.8 for atROL-D4. The quantification was performed using the calibration curve. Data acquisition and processing were performed by Analyst version 3.0 software (Sciex, multiQuant 3.0).

### 4.12. Electron Microscopic Analysis

iRPE were fixed in the modified Karnovsky’s solution (2.5% glutaraldehyde/2% PFA) at 4 °C before processing. All subsequent electron microscopy sample preparation and detection were performed in Electron Microscopy Core Facility of Sun Yat-sen College of Medical Science, Sun Yat-sen University. For scanning electron microscope (SEM) analysis, iRPE on Transwell inserts were dehydrated in a graded series of ethanol/water, transferred into 100% acetone for 15–20 min, 100% isoamyl acetate for 15–30 min. Samples were dried using the Leica EM CPD 300 (Leica Microsystems, Wetzlar, Germany), and sputter-coated with gold using the Coater Ion sputter EIKO IB-5 (EIKO, Tokyo, Japan). Finally, samples were analyzed with SEM (FEI Quanta 200, FEI company, Hillsboro, OR, USA) at 5 kV acceleration voltage using the lower secondary electron detector.

For transmission electron microscope (TEM) analysis, iRPEs on Transwell inserts were dehydrated by passing through a graded series of ethanol solutions, and embedded in epoxy resin. Then, samples were cut into ultrathin sections with an ultra-microtome, stained with 1% uranyl acetate and lead citrate, and then imaged with a transmission electron microscope (FEI company, Hillsboro, OR, USA).

### 4.13. RNA-Seq and Data Analysis

RNA-seq analysis was performed as previously described [57]. iRPE cells (about 1 × 10^6^ cells per experiment, 3 experiments) from different groups were collected in Trizol reagent (Invitrogen, Grand Island, NY, USA) and stored in a −80 °C freezer. Total RNA was extracted according to the manufacturer’s instructions. RNA quality and quantity were assessed using the Agilent 2100 bioanalyzer (Agilent Technologies, Santa Clara, CA, USA). mRNA libraries were then set up and RNA-seq was performed by Illumina Novaseq 6000 (Illumina, San Diego, CA, USA). Raw data were processed using the fastp tool (version 0.18.0) [58]. Reads containing poly-Ns, duplicate sequences, and low-quality sequences were removed to obtain high-quality clean reads. The remaining clean reads were further used in assembly and gene abundance calculation. Gene expression levels were quantified by software RSEM (v1.3.3). The fragment per kilobase of transcript per million mapped reads (FPKM) value was calculated to quantify the expression levels of each gene. Correlation analysis was performed by R. correlation of two parallel experiments. Principal component analysis (PCA) was performed with R package g models (http://www.r-project.org/). Differential expression analysis was performed by DESeq2 R package (1.18.0) between two different groups and by edgeR between two samples. The genes/transcripts with the parameter of false discovery rate (FDR) below 0.05 and absolute fold change ≥1.5 were considered differentially expressed genes (DEGs). Enrichment scores and *p* values were calculated as default parameters, and *p* < 0.05 was considered statistically significant.

### 4.14. Statistical Analysis

Data in the experimental section are expressed as mean ± SEM. Statistical analysis was performed with GraphPad Prism version 9.0. The statistical significance of difference was determined by unpaired *t*-test for two-group comparisons or ANOVA followed by Dunnett’s test for comparisons among three groups or more. *p* < 0.05 was considered statistically significant.

## Data Availability

The original contributions presented in this study are included in the article/Supplementary Materials. Further inquiries can be directed to the corresponding author.

## Supplementary Materials

The following supporting information can be downloaded at: https://www.mdpi.com/article/10.3390/ijms27135643/s1.

## Abbreviations

The following abbreviations are used in this manuscript:

AAV	Adeno-associated virus
*BEST1*	Bestrophin 1
CHX	Cycloheximide
CMV	Cytomegalovirus
CQ	Chloroquine
eGFP	Enhanced green fluorescence protein
HEK	Human embryonic kidney
hiPSCs	Human induced pluripotent stem cells
ITR	Inverted terminal repeat
kDa	kiloDalton
LCA	Leber’s congenital amaurosis
LC-MS	Liquid Chromatography-Mass Spectrometry
*LRAT*	Lecithin Retinol Acyltransferase
POS	Photoreceptor outer segments
RE	Retinyl ester
RPE	Retinal pigment epithelium
*RPE65*	Retinal pigment epithelium-specific 65
ROs	Retinal organoids
SEM	Scanning electron microscope
TEM	Transmission electron microscope
TER	Transepithelial resistance
VUS	Variants of unknown significance
WB	Western blot
WT	Wild type
