# Supplementary material for: Personalized Pathogenicity Assessment of RPE65 Gene Mutations Using Patient-Specific hiPSC-Derived Retinal Pigment Epithelium Model
Source: Int J Mol Sci. 2026 Jun 23;27(13):5643. doi: 10.3390/ijms27135643 (PMC13361107; doi:10.3390/ijms27135643)
Supplement: Supplementary file 1 [file ijms-27-05643-s001.zip › ijms-4304491-supplementary.pdf]

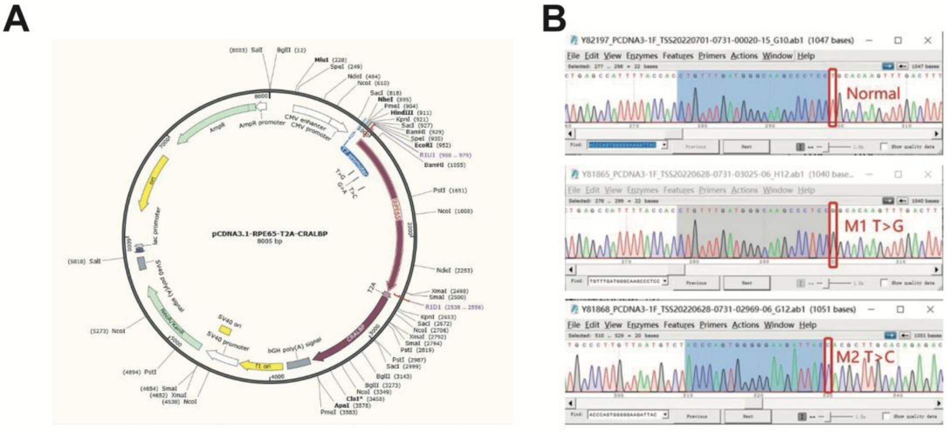

Figure S1 Construction of *in vitro* visual cycle using HEK293T cells.

(A) Profile of the *RPE65*/*CRALBP* expression vector.

(B) Sanger sequencing of expression vectors with WT *RPE65*, M1 and M2 mutant *RPE65*.

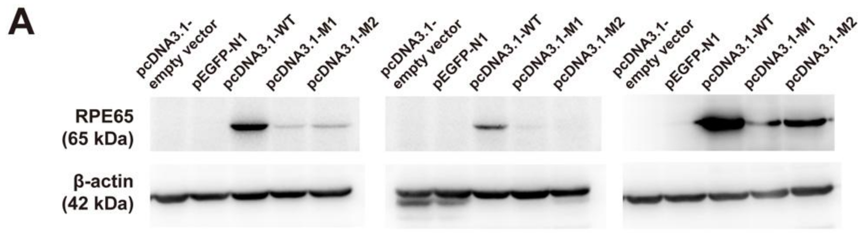

Figure S2 Validation of undetectable endogenous RPE65 expression in HEK293T cells.

(A) Western blot analysis of RPE65 protein in HEK293T cells transfected with empty vector, WT or mutant *RPE65* constructs.

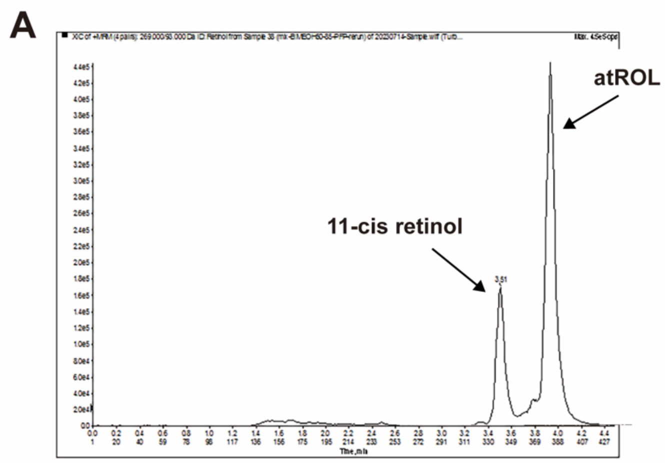

Figure S3 LC-MS analysis of *in vitro* visual cycle using HEK293T cells.
(A) Chromatogram of 11-cis ROL and atROL using standard reference.

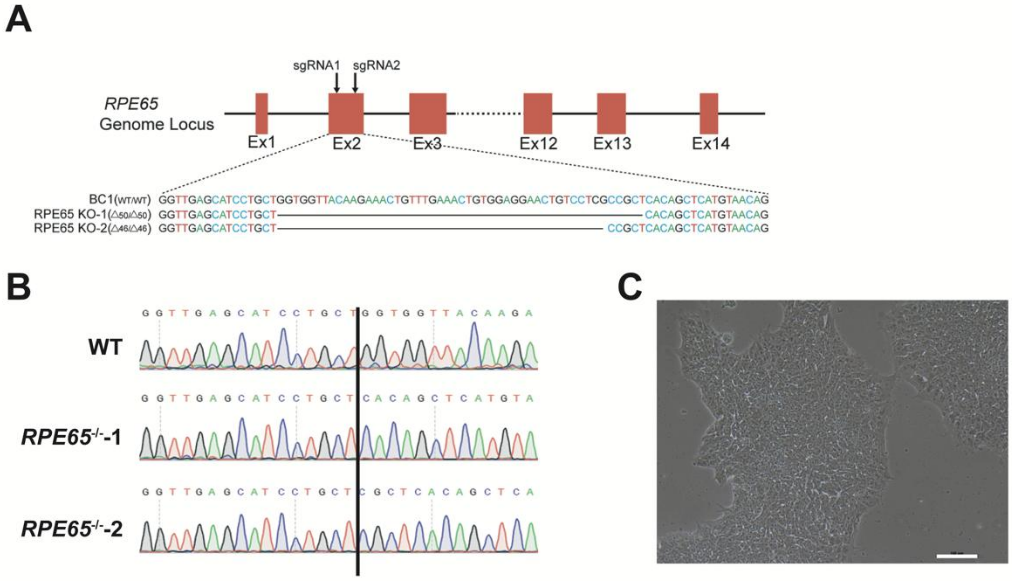

Figure S4 Construction of *RPE65*$^{-/-}$ hiPSCs

(A) Target strategy of generation of *RPE65*$^{-/-}$ hiPSCs from BC1 hiPSCs.

(B) Sanger sequencing of *RPE65*$^{-/-}$ hiPSCs.

(C) Representative bright field images of *RPE65*$^{-/-}$ hiPSCs. Scale bar = 100 μm

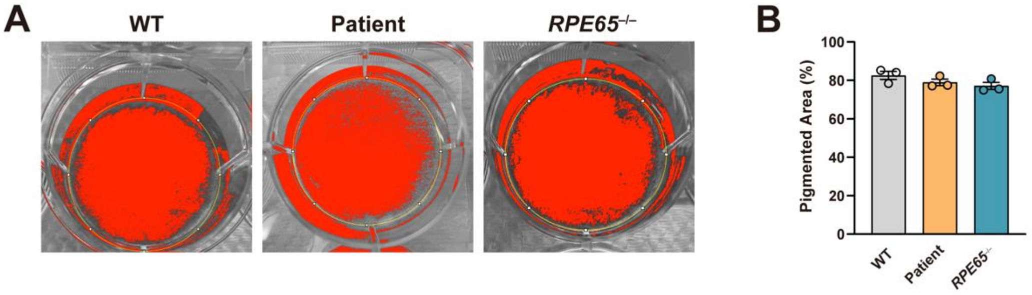

Figure S5 High efficiency of RPE differentiation from hiPSCs.

(A) Representative macroscopic observation of the culture dishes after 35 days of differentiation.

(B) Quantification of the pigmented area in culture dishes. Data represent mean ± SEM.

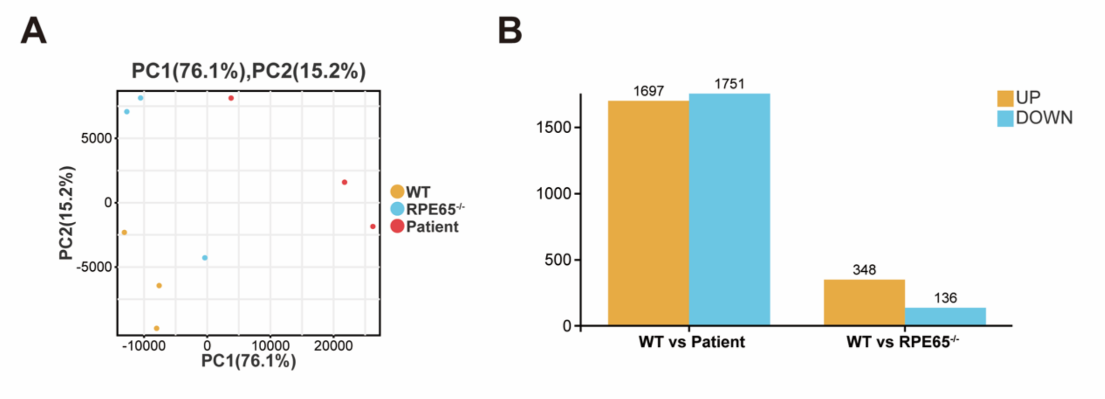

Figure S6 RNA-seq analysis of three types of iRPE cells.

(A) Principal Component Analysis (PCA) of three types of iRPE cells.

(B) Numbers of DEGs among three types of iRPE cells.

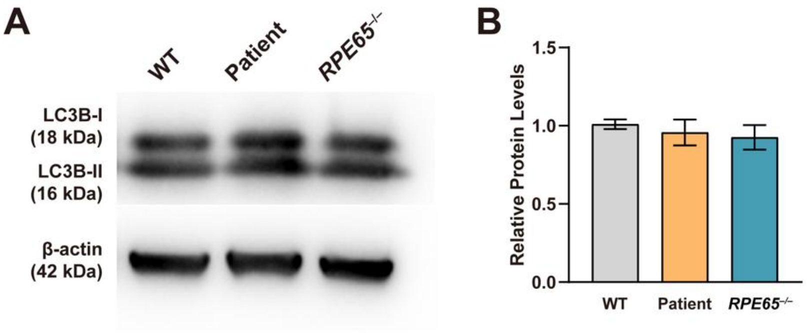

Figure S7 LC3 protein level in iRPE cells.

(A) Western blot analysis of LC3B in WT, patient-specific and *RPE65*$^{-/-}$ iRPE cells.

(B) Quantification of LC3B protein levels in WT, patient-specific and RPE65-/- iRPE cells. Data represent mean ± SEM.

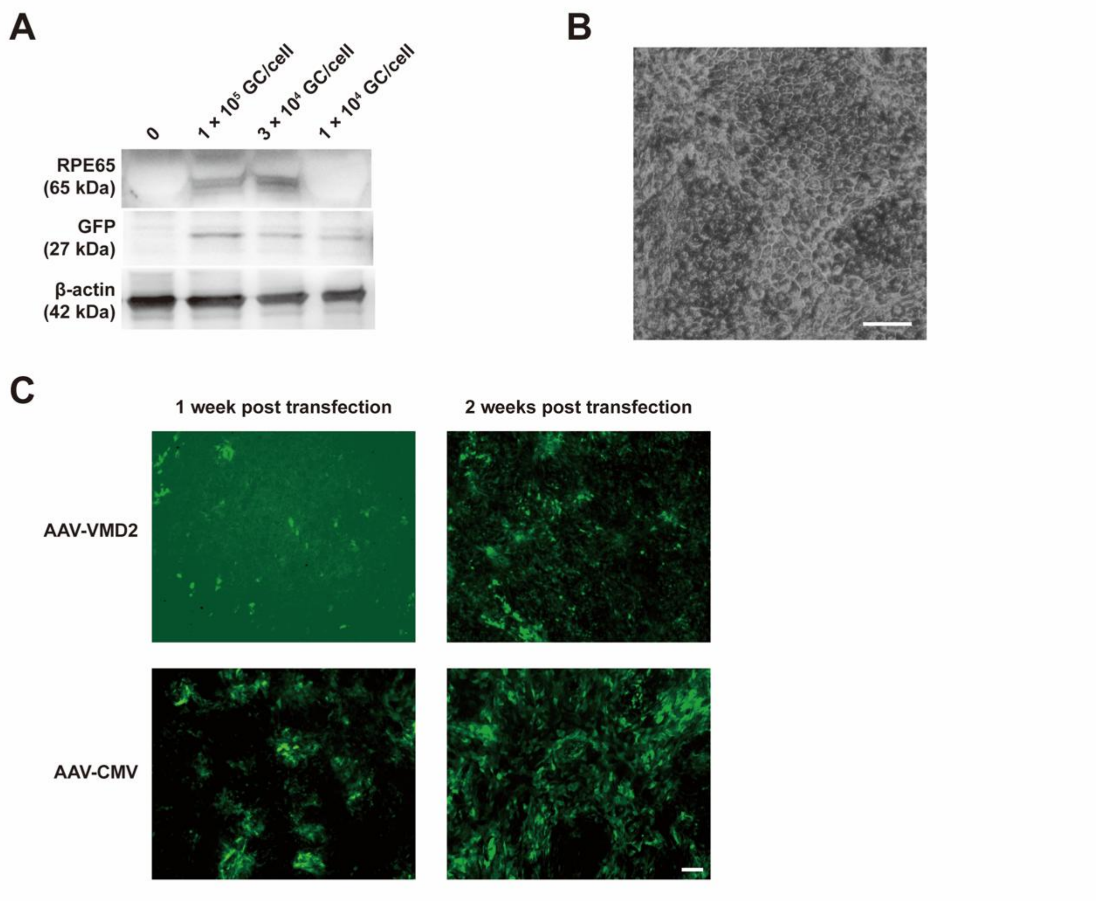

Figure S8 Construction of *in vitro* visual cycle using HEK293T cells.

(A) Western blot analysis for RPE65 and GFP in patient-specific iRPE cells with or without AAV-VMD2-RPE65 in different GC/cell.

(B) Representative microscopic images of patient-specific iRPE cells with AAV-VMD2-RPE65 treatment in week 8. Scale bar = 50 μm

(C) Representative live images for GFP in patient-specific iRPE cells 1 week and 2 weeks post transduction of AAV-RPE65 with different promoters. Scale bar = 100 μm

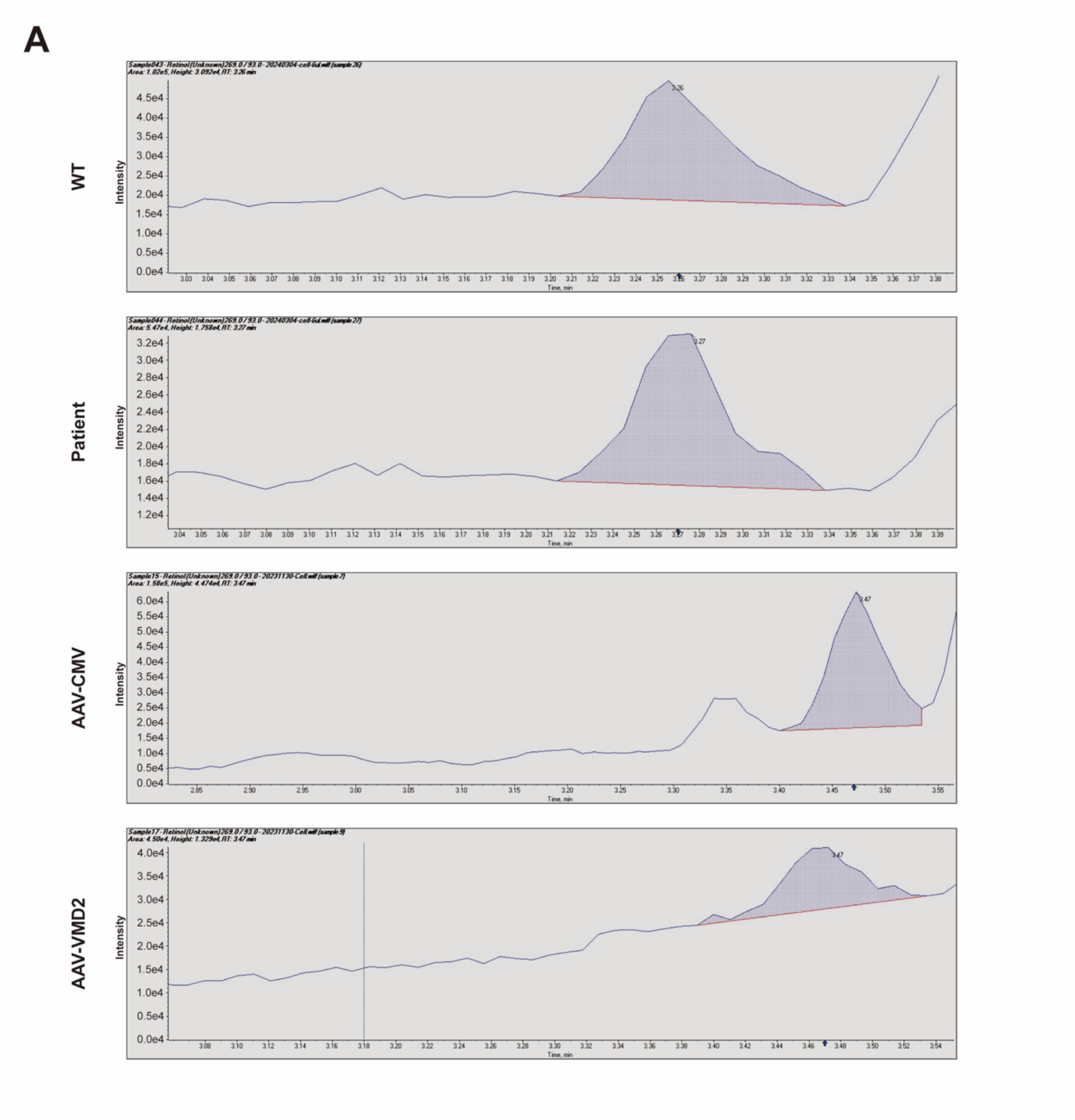

Figure S9 LC-MS analysis of patient-specific iRPE cells with AAV treatment.

(A) Chromatogram of 11-cis ROL in WT iRPE cells, patient-specific iRPE cells with or without the treatment of AAV-RPE65 with different promoters.

Supplementary Table S1: List of primers used in this study

NO	Primer	Sequence
1	RPE65 sgRNA1-F	F 5'CACCGTACATGAGCTGTGAGCGGCG 3'
2	RPE65 sgRNA1-R	F 5'AAACCGCCGCTCACAGCTCATGTAC 3'
3	RPE65 sgRNA2-F	F 5'CACCGAGTTTCTTGTAACCACCAGC 3'
4	RPE65 sgRNA2-R	F 5' AAACGCTGGTGGTTACAAGAAACTC 3'
5	RPE65-KO-VF1	F 5' CTCCCAGGTGCTTGTCACTG 3'
6	RPE65-KO-VR1	F 5' CTAGACCGGCAGGAGTGAAC 3'
7	RPE65-F	F 5' ATGTCTATCCAGGTTGAGCATC 3'
8	RPE65-R	F 5' TCAAGATTTTTGAACAGTCCA 3'
9	LRAT-F	F 5'AGGGATGAAGAACCCCATGC 3'
10	LRAT-R	F 5' TTAGCCAGCCATCCATAGGAAG 3'
11	CRALBP-F	F 5' CAACATGTCAGAAGGGGTGG 3'
12	CRALBP-R	F 5'TCAGAAGGCTGTGTTCTCAGC 3'
13	RPE65-qPCR-F	F 5'GCCCTCCTGCACAAGTTTGACTTT 3'
14	RPE65-qPCR-R	F 5'AGTTGGTCTCTGTGCAAGCGTAGT 3'
15	GAPDH-qPCR-F	F 5'TCGTGGAAGGACTCATGACC 3'
16	GAPDH-qPCR-R	F 5'AGGCAGGGATGATGTTCTGG 3'

Supplementary Table S2 List of antibodies used in this study

Antibody	Species	Brand	Concentration
anti-ZO-1	mouse	Thermo Fisher Scientific	1: 400 for IHC
anti-BEST1	mouse	Abcam	1: 500 for IHC
anti-RPE65	mouse	Abcam	1: 200 for IHC;<br>1: 1000 for WB;
anti-CRALBP	mouse	Abcam	1: 500 for IHC
anti-LC3B	rabbit	Abcam	1: 200 for IHC;<br>1: 2000 for WB;
anti-β-actin	mouse	Beyotime	1: 1000 for WB
anti-Mouse, Alexa Fluor 555	donkey	Invitrogen	1: 1000 for IHC
anti-Rabbit, Alexa Fluor 555	donkey	Invitrogen	1: 1000 for IHC
anti-Rabbit, Alexa Fluor 488	donkey	Invitrogen	1: 1000 for IHC
anti-Rabbit, Alexa Fluor 647	donkey	Invitrogen	1: 1000 for IHC
